# The Role of Marital Status in the Association between Benzodiazepines, Psychotropics and Injurious Road Traffic Crashes: A Register-Based Nationwide Study of Senior Drivers in Sweden

**DOI:** 10.1371/journal.pone.0086742

**Published:** 2014-01-29

**Authors:** Kristina Johnell, Lucie Laflamme, Jette Möller, Joel Monárrez-Espino

**Affiliations:** 1 Aging Research Center, Karolinska Institutet and Stockholm University, Stockholm, Sweden; 2 Department of Public Health Sciences, Karolinska Institutet, Stockholm, Sweden; University of Manitoba, Canada

## Abstract

**Background:**

Among senior drivers, benzodiazepines (BZDs) have a documented effect on the risk of road traffic crashes (RTCs). It remains unclear however if BZDs play the same role when considering marital status. Therefore, we aimed to investigate the role of marital status in the association between BZD use and injurious RTCs among senior drivers.

**Methods:**

Matched case-control study based on five national Swedish registers (n = 154 225). Cases comprised the first non-alcohol-related injurious RTC sustained by drivers aged 50–80 years from July 2005 to December 2009 and controls included registered residents with a valid license who did not crash during that period. Four controls were matched to each case by sex, age and place of residence. Conditional logistic regression analysis for injurious RTC was performed with adjustment for occupation and number of medications. The main exposure was dispensation of BZDs, alone or in combination with other psychotropic medications, 1–30 days prior to the crash date stratified by marital status.

**Results:**

BZD use, alone or in combination with other psychotropic medications, increased the risk of being involved in an RTC (BZD only: adjusted OR: 1.26, 95% CI: 1.17–1.36; BZDs and other psychotropics: adjusted OR: 1.25, 95% CI: 1.12–1.41). Compared to married drivers, those divorced (1.48, 1.43–1.53) and widowed (1.54; 1.45–1.63) had higher adjusted ORs**.** Marital status modified the association between BZDs and RTCs, particularly among younger male drivers.

**Conclusions:**

Both BZDs and marital status independently affect the risk for senior drivers to be involved in an RTC. However, marital status plays a role in the association between BZD use and RTCs and this may have implications for targeting risk populations for RTCs among senior drivers.

## Introduction

The elderly population is increasing worldwide with a large proportion living an active and autonomous life over a long period of time. It has been forecasted that an increasing number of pensioners will possess a driving licence and that both male and female drivers will drive more and longer than their present-day counterparts [1], [2]. This will have implications for road safety that will influence the agenda for preventing road traffic related injuries.

Studies indicate that older drivers might be involved in fewer crashes globally, but due to cognitive reasons [3], [4], they are over-represented in some specific types of crashes; their higher frailty also results in higher risks of sustaining severe consequences when involved in a road traffic crash (RTC) [1], [2].

However, older drivers are not a homogeneous group and their likelihood of being involved in RTCs can vary depending on a variety of individual factors, not least their socio-demographic characteristics. From among those, marital status is one that to our knowledge has received little attention [5]–[8]. Yet, just as there is an acknowledged protective effect of being married on health in general, there are several mechanisms that may lead to differences in the risk of crashing among older people based on their marital status. A first type of mechanism is that of differential exposure to road traffic (e.g. mode of transport, driving patterns, distance driven, road traffic environment, and vehicle conditions) that affects directly how likely it is for senior drivers to be involved in a RTC [9], [10]. Another type of mechanism is differences in how likely it is to get injured once involved in a crash, e.g., related to the type of vehicle one drives (vehicle crashworthiness). Finally, it is also likely that remote individual factors come into play, affecting both the risk of crashing and that of being severely injured, such as differences in lifestyle or in physical and mental health conditions. Those, in turn, can vary depending on the drivers’ marital status.

In this study, attention was paid to the use of psychotropic medications by senior drivers, which can influence the risk of crashing by producing side effects that negatively impact on driving performance, but whose prescription is also related to the drivers’ age, health condition, and marital status [8], [11]–[14]. We focused on benzodiazepines (BZDs), a well-established risk factor for RTCs [15]–[19]. These drugs can cause cognitive and psychomotor impairment and sedation [20], above all among the elderly [21], where they are commonly used [22].

The aim of this study was to investigate the role of marital status in the association between BZD use and the involvement in injurious RTCs among senior drivers.

## Methods

### Ethics Statement

The study was approved by the Regional Ethical Review Board in Stockholm, Sweden (dnr 201/865-31/2). All data were extracted from registers located at the Swedish Transport Agency (www.transportstyrelsen.se), Statistics Sweden (www.scb.se) and the Swedish National Board of Health and Welfare (www.socialstyrelsen.se). Data are recorded in these national registers without written consent. We only analyzed de-identified data.

### Study Design

We performed a Swedish nationwide, population-based matched case-control study with a follow-up from July 1, 2005 to December 31, 2009. The source population included all registered residents aged 50 to 80 years old [23]. The Swedish Traffic Accident Data Acquisition (STRADA) register, administered by the Swedish Transport Agency, was used to identify cases of drivers involved in an injurious car, truck or bus crash. Only the first event during the study period was included. Drivers reported by the police as suspected of being under the influence of alcohol at the time of the crash were excluded, as this is known to be a major determinant for RTCs [24], [25]. Information regarding the date and place of the crash, passenger position (i.e. driver *vs.* passenger), main consequence of the crash (e.g. severe injury or death), and other crash characteristics was extracted from STRADA.

The Register of the Total Population and the National Driver’s License Register (administered by the Swedish Transport Agency) were used to randomly select four controls from the general population holding a driving license authorizing them to drive at least a car and who were not involved as drivers in a RTC during the study period. Controls were matched to each case by age (year and month of birth), sex, and place of residence by using eight geographic areas. Once controls were assigned, they were censored so that they could not be allocated to any other case of equal characteristics. In total, the study includes 30 845 cases and 123 380 matched controls.

Individual data on medications among cases and control subjects were collected from the Swedish Prescribed Drug Register (SPDR). The SPDR contains information on all prescribed and dispensed medications for the entire Swedish population since 1 July 2005 including the generic names of drug substances according to the Anatomical Therapeutic Chemical (ATC) classification [26] and the date of prescription and dispensation. However, the register lacks information on indication for treatment, drugs bought over-the-counter, and medications administered during hospitalizations.

### Benzodiazepine Use

Dispensed drug prescriptions of the following BZDs were identified using their specific ATC code in the SPDR: Diazepam (N05BA01), nitrazepam (N05CD02), flunitrazepam (N05CD03), clonazepam (N03AE01), oxazepam (N05BA04), lorazepam (N05BA06), alprazolam (N05BA12), triazolam (N05CD05) and midazolam (N05CD08) [22].

Although we assessed four exposure periods (1–15, 1–30, 1–90 and 1–120 days prior to the index date), the results refer to BZDs dispensed within the 1–30 day period before the index date in the attempt to assure actual exposure. The following four exposure categories were defined:

1. Dispensed no BZD or any other psychotropic medication (ATC code N05A–antipsychotics, N05B–anxiolytics, N05C–hypnotics/sedatives, and N06A–antidepressants) (reference category),
2. Dispensed at least one BZD but no other psychotropic medication,
3. Dispensed at least one BZD and at least one other psychotropic medication, and
4. Dispensed at least one psychotropic medication but no BZD.

### Marital Status

Marital status was derived from the Register of the Total Population, which contains individual socio-demographic characteristics on all Swedish residents, including marital status, sex, age and place of residence. Marital status was defined on the basis of the most recent marital status recorded prior to the index date. The following four categories were available: married, divorced, widowed and unmarried. However, the results for the category unmarried are not presented, as this group comprise both single people and cohabiting individuals without registered partnership or marriage [27] potentially leading to classification bias.

### Potential Confounders

Besides age, sex and place of residence, which were used to match the cases to controls, number of medications [23] was also taken into account in the analyses as a proxy for overall co-morbidity [28]–[31]. Number of medications was defined as number of different medications dispensed in the exposure period (1–30 days) based on the full five-level ATC code, excluding the dispensations of BZDs. Further, based on data from the National Patient Register, we calculated Charlson Comorbidity Index as an alternative proxy for co-morbidity.

Information regarding the occupation of the driver was assessed based on the the Longitudinal Integration Database for Health Insurance and Labor Market Studies (abbreviated as LISA in Swedish) and adjusted for in the analyses. Occupations were grouped into three major categories: Professionals (e.g. legislators, senior officials, managers and professionals), technicians (e.g. technicians and associate professionals), and skilled workers (e.g. skilled agricultural/fishery workers, craft/trade workers, plant/machine operators/assemblers and elementary occupations).

### Statistical Analyses

Socio-demographic characteristics of the senior drivers were described using frequencies and proportions to compare cases and controls in terms of marital status, matched variables and occupation as a potential confounder. The frequencies of the four previously defined exposure categories of BZDs and other psychotropics were compared between cases and controls.

The distribution of injurious RTCs was graphically presented by age and sex.

To assess the effect of BZD use and marital status on RTCs, conditional logistic regression was used to compute crude and adjusted odds ratios (ORs) with 95% confidence intervals (CIs). Firstly, crude and adjusted odds for injurious RTCs were estimated using marital status (married drivers as reference group) and BZD/psychotropic dispensations within 1–30 days prior to the index date (non-exposed as reference group) as exposure variables.

Then the ORs, adjusted for occupation and number of different medications, were estimated for injurious RTCs among drivers exposed to BZDs within 1–30 days prior to the index date, alone or in combination with other psychotropics. The results were stratified by drivers’ sex, age group (50–65 and 66–80 years), and marital status (married, divorced and widowed). These stratified analyses focused only on the exposure categories that comprised BZDs.

Data compilations and statistical analyses were performed using SAS software version 9.2 (SAS Institute Inc., North Carolina).

## Results

There were 30,845 first non-alcohol related injurious RTCs involving senior drivers aged 50–80 years old during the study period. At least two vehicles were involved in most of these RTCs (85.6%). Collisions occurred mainly during daylight (71.3%) and under clear weather conditions (78.8%). In 16% of the crashes, at least one injured person required hospital care, and in 2.4% at least one person died.

**Figure 1:**
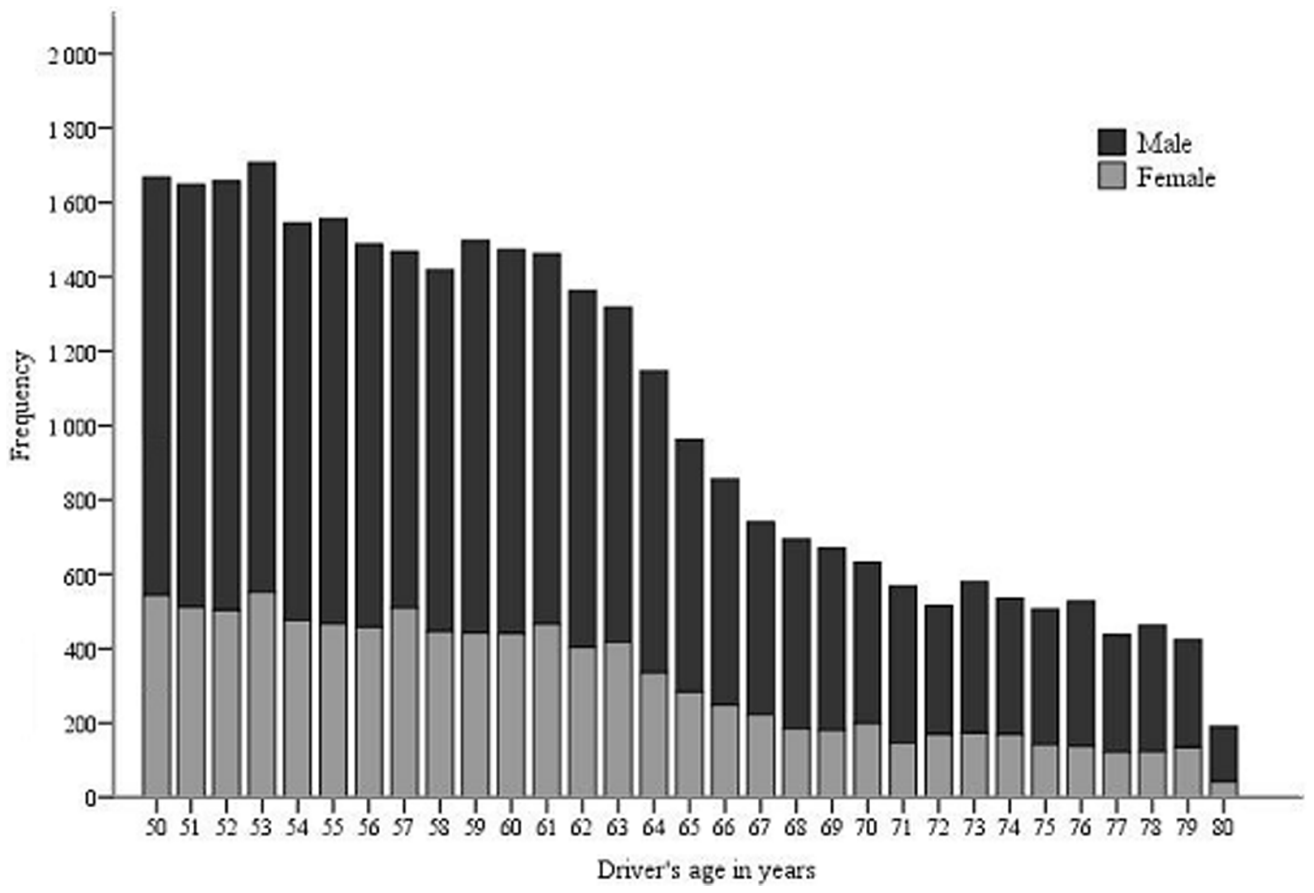
Frequency of injurious road traffic crashes (cases) by drivers’ age and sex among senior drivers, Sweden 2005–2009.

Figure 1 shows that the number of RTCs among senior drivers decreased with increasing age, both among male and female drivers. A more pronounced decline could be observed after the age of retirement (65 years in Sweden).

Table 1 shows that the majority of crashes involved male drivers (69.6%), drivers aged 50–65 years (71.5%) and most crashes occurred in southern and central Sweden (90.4%), where the large majority of the Swedish population lives. Compared with the controls, there were slightly lower proportions of cases classified as professionals or technicians, and a higher proportion of skilled workers. With regard to marital status, cases were less likely to be married (55.4 *vs.* 63%), but more likely to be divorced (23.2 *vs.* 17.4%) or widowed (6.4 *vs.* 4.8%). Cases were also more likely than the controls to be exposed to BZDs within 1–30 days prior to the index date (alone or in combination with other psychotropics) (p<0.001).

**Table 1:** Distribution of socio-demographic characteristics and dispensation of benzodiazepines (BZDs) and psychotropic medication 1–30 days prior to the index date for cases and controls among senior drivers aged 50–80 years, Sweden 2005–2009.

Characteristics		Frequency (%)		P-value
		Cases n = 30845	Controls n = 123380	
Sex	Men	69.6	69.6	Matched
	Women	30.4	30.4	
Age group	50–65 years	71.5	71.5	Matched
	66–80 years	28.5	28.5	
Residence place^1^	North	9.6	9.6	Matched
	Central	43.3	43.3	
	South	47.1	47.1	
Occupation	Professionals	21.0	23.5	<0.001
	Technicians	31.7	34.5	
	Skilled workers	33.0	28.3	
	Unknown/missing	14.3	13.7	
Marital status	Married	55.4	63.0	<0.001
	Divorced	23.2	17.4	
	Widowed	6.4	4.8	
	Unmarried	14.7	14.1	
	Unknown/missing	0.2	0.6	
BZDs and psychotropics^2^	None	92.6	94.5	<0.001
	BZDs only	3.0	2.2	
	BZDs+other psychotropics	1.4	1.0	
	Other psychotropics only	2.9	2.3	

1 Matched by using eight geographic areas, but summarized here for presentation purposes.

2 Dispensed ≥1 BZD and/or ≥1 other psychotropic medication (N05A-antipsychotics, N05B- anxiolytics, N05C-hypnotics/sedatives, and N06A-antidepressants) 1–30 days prior to the index date.

Table 2 shows crude and adjusted ORs for injurious RTCs by drivers’ marital status and dispensation of BZDs and other psychotropic medications. Compared with married drivers, the divorced and widowed drivers showed significantly higher crude ORs for injurious RTCs (OR: 1.51, 95% CI: 1.46–1.55 and OR: 1.56, 95% CI 1.48–1.66, respectively), and the magnitude of the effect remained virtually unchanged after adjusting for occupation and number of medications (adjusted OR: 1.48, 95% CI: 1.43–1.53 and adjusted OR: 1.54, 95% CI: 1.45–1.63, respectively).

**Table 2:** Matched^1^ crude and adjusted ORs with 95% CI for injurious road traffic crashes by marital status and benzodiazepine (BZD) and psychotropic medication use 1–30 days prior to the index date among senior drivers aged 50–80 years, Sweden 2005–2009.

Exposure	Exposed, n		OR (95% CI)	
	Cases	Controls	Crude	Adjusted^2^
Marital Status				
Married	17103	77669	Reference	Reference
Divorced	7143	21522	1.51(1.46–1.55)	1.48(1.43–1.53)
Widowed	1981	5944	1.56(1.48–1.66)	1.54(1.45–1.63)
BZDs and psychotropics^3^				
None	28566	116648	Reference	Reference^4^
BZDs only	929	2729	1.39(1.29–150)	1.26(1.17–1.36)
BZDs+otherpsychotropics	447	1180	1.55(1.39–1.73)	1.25(1.12–1.41)
Otherpsychotropics only	903	2823	1.30(1.21–1.41)	1.16(1.07–1.26)

1 By sex, age, and place of residence (four controls per case).

2 Adjusted for occupation and number of different dispensed medications within 1–30 days prior to the index date.

3 Dispensed ≥1 BZD and/or ≥1 other psychotropic medication (N05A-antipsychotics, N05B- anxiolytics, N05C-hypnotics/sedatives, and N06A-antidepressants) 1–30 days prior to the index date.

4 Adjusted for marital status, occupation and number of medications within 1–30 days prior to the index date (BZD dispensations were subtracted).

Dispensation of at least one BZD 1–30 days prior to index date alone or in combination with at least one other psychotropic medication significantly increased the risk of being involved in an injurious RTC, even after adjustment for marital status, occupation, and number of medications (BZD only: adjusted OR: 1.26, 95% CI: 1.17–1.36; BZDs and psychotropics: adjusted OR: 1.25, 95% CI: 1.12–1.41).

Table 3 presents adjusted ORs for injurious RTCs in senior drivers exposed to BZDs only or in combination with other psychotropic medication within 1–30 days prior to the index date stratified by sex, age group, and marital status. Statistically significant associations were seen only among drivers dispensed BZDs alone. In those aged 50–65 years, both married men and women showed significantly increased ORs when driving under the influence of BZDs, particularly women (adjusted OR: 1.66, 95% CI: 1.28–2.15). However, only divorced men, and not divorced women, had a higher risk of crashing while under the influence of these drugs (adjusted OR: 1.85, 95% CI: 1.17–2.91). The small number of widowed drivers aged 50–65 years precluded statistical analyses of this group. Among those aged 66–80 years, married drivers were also more likely to be involved in injurious RTC after being dispensed BZDs only, but statistical significance was reached only among male drivers (adjusted OR: 1.28; 95% CI: 1.03–1.61). Divorced or widowed drivers aged 66–80 years who used BZDs did not show a significantly increased risk of involvement in injurious RTCs regardless of sex.

**Table 3:** Matched adjusted ORs with 95% CI for injurious road traffic crashes in senior drivers exposed to benzodiazepines (BZD), alone or in combination with psychotropic medications, within 1–30 days prior to the index date stratified by sex, age group, and marital status, Sweden 2005–2009.

Drivers’ age groupand marital status	Matched adjusted OR (95% CI)^1^			
	BZDs only^2^		BZDs+other psychotropics^3^	
	Men	Women	Men	Women
50–65 years				
Married	1.29 (1.04–1.59)	1.66 (1.28–2.15)	1.17 (0.82–1.66)	1.13 (0.79–1.62)
Divorced	1.85 (1.17–2.91)	1.08 (0.74–1.58)	1.61 (0.92–2.82)	1.17 (0.69–1.98)
Widowed	N/A	0.50 (0.09–2.73)	N/A	N/A
66–80 years				
Married	1.28 (1.03–1.61)	1.37 (0.92–2.06)	1.15 (0.75–1.77)	0.94 (0.44–2.02)
Divorced	1.21 (0.68–2.16)	0.98 (0.49–1.92)	1.86 (0.59–5.84)	2.15 (0.80–5.72)
Widowed	1.97 (0.70–5.50)	1.33 (0.86–2.06)	1.31 (0.19–8.95)	0.43 (0.17–1.09)

1 Matched by age, sex and residence place (four controls per case); adjusted for occupation and number of medications dispensed within 1–30 days prior to the index date.

2 Exposed to at least one BZD but no other psychotropic medications.

3 Exposed to at least one BZD and at least one other psychotropic medication (N05A-antipsychotics, N05B- anxiolytics, N05C-hypnotics/sedatives, and N06A-antidepressants).

N/A: Not available (unable to estimate due to small subgroup).

## Discussion

### Main Findings

In this nationwide study, we have shown that the risk of being involved in an injurious RTC among senior drivers differs considerably depending on their marital status. Being married was associated with substantially lower risks of being involved in RTCs compared with being a widow or divorced. This finding is much in line with earlier studies on risk factors for RTCs and injuries conducted on different age segments of the population [5], [7], [12], [32]–[34]. Although the protective effect of being married seems to remain in older male and female drivers, the factors that come into play remain to be determined. As mentioned earlier, there are reasons to believe that there are pronounced differences in the health condition of senior drivers of different marital status [35], [36]. Their medication is one way to approach this question [37], [38].

As expected, the risk for older drivers to crash increased with exposure to BZDs [8], [11], [13], [14], either with or without concomitant use of other psychotropic medications. However, the association varied across categories of marital status. Our results suggest that marital status modifies the risk of crashing when drivers use BZDs. The association seems to be more pronounced among those aged 50–65 years. There were also important differences between male and female drivers, where marital status seemed to matter more for men than for women in the association between use of BZDs and RTC. This result may be partly explained by the smaller number of female drivers in our study. Also, divorced men might be more confident and less careful when driving after intake of BZDs compared to divorced women. A plausible explanation for the stronger association between BZD use and RTCs among younger senior drivers could be the existence of other risk factors in this age group including greater risk taking on the road [32]. It could also reflect a lower effect of BZDs among the older drivers aged 66–80 years, [39] who might have developed adaptation to these medications [40].

### Strengths and Limitations

The main strength of this study lies in the linking of individual information on police-reported RTCs, which safeguards that the injured person actually was the driver of the car that crashed, to the SPDR and various other health and administrative registers. These registers are continually updated and cover the whole Swedish population. Another strength is the accuracy and completeness of the crash data, which were collected by highly trained policemen at the scene of the crash. We thus believe that the results can be generalized to other settings with similar motor traffic systems and driving practices among older persons.

One potential limitation of the study is that although RTCs resulting in serious injuries are recorded in the STRADA register, RTCs causing minor injuries might have gone undetected by the police. The fact that we used crash involvement instead of responsibility is a potentially relevant issue that could have biased the results if the non-responsible drivers were not a random sample of the population [41]. However, when restricting the analyses to the single-vehicle crashes where drivers could be presumed responsible for the event (4 445 cases), as this avoids misclassification errors, the adjusted effects for BZDs (1–30 prior to the crash) were of even higher magnitude and significance (BZDs only: OR: 1.66, 95% CI: 1.38–1.99; BZDs+other psychotropics: OR: 2.53, 95% CI: 1.97–3.25; and psychotropics excluding BZDs OR: 1.46, 95% CI: 1.21–1.77), as were the effects for the marital status categories when compared with married drivers (divorced: OR: 1.60, 95% CI: 1.47–1.74; widowed: OR: 1.82, 95% CI: 1.58–2.10).

Since the exposure to medications was assessed by using register information, thereby reducing the risk of recall bias, we may have overestimated the adherence rate because dispensation data is not synonymous to actual intake [42]. This could be of importance if the adherence rate varied between cases and controls depending on the number of dispensed medications. Indeed, increasing number of medications have been associated with higher non-adherence [43], though no evidence for BZDs is available in this context. If this was the case, the effects seen here would then be underestimated.

We used number of medications as a proxy for overall comorbidity [28]–[31]. This measure may not adequately capture the entire health status of an individual. However, we also made separate analyses with adjustments for Charlson Comorbidity Index as a proxy for comorbidity. In those models, we found that adjustment for Charlson Comorbidity Index yielded less conservative results than number of medications. There was also a risk of collinearity when including both Charlson Comorbidity Index and number of medications within the same model.

The use of the 30 day period prior to the index date to define BZD dispensation aimed at assessing current exposure. We did not stratify the results using other more refined BZD classifications (e.g. anxiolytics *vs.* hypnotics, short- *vs.* long-acting pharmacologic activity, and recent *vs.* chronic use), which have been related to differential risks of crashing [40], as the study focused on exploring the role of marital status in the association between BZD use and crash occurrence rather than on differences in BZD use. Yet, it is true that misclassification of the exposure could have occurred in drivers who received dispensations more than 30 days prior to the index date that were still taking the medication within the 1–30 days exposure period set. However, when we used the 1–90 day definition, we observed similar odds across the marital status categories, but with decreasing ORs for BZD use with longer exposure periods, as expected.

Marital status was assessed by using the closest reported marital status of the driver prior to the index date according to the Register of the Total Population, which is updated yearly in December. This means that the drivers’ marital status could have changed before the index date, leading to misclassification. For instance, a married person according to the register in December 2007 who became widow in March 2008 would have been classified as married if the index date was between April and November 2008. Nonetheless, it is unlikely that this possible misclassification was differential between cases and controls.

Another potential issue concerning marital status relates to the lack of reliable information about single people. The register data for the unmarried group cannot differentiate between single status and co-habiting without registered partnership or marriage. Therefore, we excluded the unmarried group from the analyses to avoid drawing false conclusions regarding this group.

Finally, this study did not offer the opportunity to compare driving frequency or driving distance between cases and controls. Instead we had to rely on license endorsement as a surrogate. This could have led to an overestimation of the effect if controls were less likely to drive compared with the cases, especially in the older age groups. However, since drivers tend to surrender their driving license as they age [44], the controls with driving license that were matched by age were likely to represent the segment of the population that still drives, particularly among those older than 65 years.

## Conclusions

The impact of use of BZDs and other psychotropic medication on the risk of injurious crash involvement was replicated in this study of senior drivers. In addition, being married was found protective for the risk of RTC compared with being divorced or widowed, with the latter being associated with the highest risk of crashing.

Differences on the risk of crashing while using BZDs varied across marital status and were noteworthy in male and female drivers from 50 years up to the age of retirement (65 years); thereafter, the effect was statistically reliable only among married men. Our results highlight the interaction between BZDs and marital status and may have implications for targeting risk populations for RTCs among senior drivers.
